# Exploring the Impact of the COVID-19 Pandemic on Twitter in Japan: Qualitative Analysis of Disrupted Plans and Consequences

**DOI:** 10.2196/49699

**Published:** 2024-04-01

**Authors:** Masaru Kamba, Wan Jou She, Kiki Ferawati, Shoko Wakamiya, Eiji Aramaki

**Affiliations:** 1 Division of Information Science Graduate School of Science and Technology Nara Institute of Science and Technology Ikoma Japan; 2 Institute of Industrial Science The University of Tokyo Tokyo Japan

**Keywords:** COVID-19, natural language processing, NLP, Twitter, disrupted plans, concerns

## Abstract

**Background:**

Despite being a pandemic, the impact of the spread of COVID-19 extends beyond public health, influencing areas such as the economy, education, work style, and social relationships. Research studies that document public opinions and estimate the long-term potential impact after the pandemic can be of value to the field.

**Objective:**

This study aims to uncover and track concerns in Japan throughout the COVID-19 pandemic by analyzing Japanese individuals’ self-disclosure of disruptions to their life plans on social media. This approach offers alternative evidence for identifying concerns that may require further attention for individuals living in Japan.

**Methods:**

We extracted 300,778 tweets using the query phrase *Corona-no-sei* (“due to COVID-19,” “because of COVID-19,” or “considering COVID-19”), enabling us to identify the activities and life plans disrupted by the pandemic. The correlation between the number of tweets and COVID-19 cases was analyzed, along with an examination of frequently co-occurring words.

**Results:**

The top 20 nouns, verbs, and noun plus verb pairs co-occurring with *Corona no-sei* were extracted. The top 5 keywords were *graduation ceremony*, *cancel*, *school*, *work*, and *event*. The top 5 verbs were *disappear*, *go*, *rest*, *can go*, and *end*. Our findings indicate that education emerged as the top concern when the Japanese government announced the first state of emergency. We also observed a sudden surge in anxiety about material shortages such as toilet paper. As the pandemic persisted and more states of emergency were declared, we noticed a shift toward long-term concerns, including careers, social relationships, and education.

**Conclusions:**

Our study incorporated machine learning techniques for disease monitoring through the use of tweet data, allowing the identification of underlying concerns (eg, disrupted education and work conditions) throughout the 3 stages of Japanese government emergency announcements. The comparison with COVID-19 case numbers provides valuable insights into the short- and long-term societal impacts, emphasizing the importance of considering citizens’ perspectives in policy-making and supporting those affected by the pandemic, particularly in the context of Japanese government decision-making.

## Introduction

### Background

The spread of COVID-19 has become a global pandemic, significantly affecting social and economic sectors worldwide [1]. In the early stages of the pandemic, health authorities recommended social distancing to control the spread of the virus, reduce cases, and avoid overwhelming health care facilities [2-4]. Each country had its own strategy for dealing with COVID-19. A survey conducted across 6 countries illustrated the public’s perception of measures taken in response to COVID-19 [5]. Other surveys have been conducted in the United Kingdom and European countries to aid interdisciplinary research on public health, particularly regarding COVID-19 [6]. Different results were observed owing to social distancing policies, which affected several aspects of life, including economic activities [7] and consumer behavior, such as drops in mobility [8]. Concerns about cybersecurity risks were also raised, as companies might not have been prepared for adequate work-from-home options for employees [9]. The association between implementing some mitigation policies in response to COVID-19 and the outcomes regarding public mobility were noted [10], one of which was also observed in Japan.

After the government confirmed the first COVID-19 case in Japan on January 16, 2020, the number of cases quickly escalated within 3 months, leading to the declaration of a state of emergency to prevent the further spread of the infection. This measure significantly impacted the daily routines and social lives of Japanese residents, forcing them to refrain from going out, close schools, work from home, and be restricted from visiting crowded locations such as department stores and movie theaters. The first state of emergency effectively reduced the number of COVID-19 cases [11], albeit at a high cost to public mental well-being, education quality, and the economy. Furthermore, the number of cases quickly bounced back, peaking at 1762 new daily cases after the state of emergency was lifted, an increase from the peak of 701 new daily cases during the first wave [11]. These numbers suggest that the government was confronted with the dilemma of mitigating the social and economic impact of the lockdown and stopping the spread of COVID-19 [12]. Due to the fluctuations in COVID-19 cases, the government declared other states of emergency, recognizing the profound and deeply rooted impact the COVID-19 lockdown could have on societal and economic levels.

There have been various investigations into the states of emergency. For instance, studies have predicted SARS-CoV-2 infections using state-space models [13] and examined their impact on mental health [14]. In the aspect of mobility, studies have shown the suppression of social activities of the masses [15]. The tourism industry was among the hardest hit sectors, and the arrival of visitors decreased by 93% by March 2020 [16]. Statistics also show that Japan’s gross domestic product in 2020 decreased by 4.28%, indicating a substantial impact on the economy [17]. Interestingly, the unemployment rate only slightly increased to 2.8% in 2020, but started declining by 2022 (2.64%), following the gradual recovery of the gross domestic product (2.14% growth by 2021 and 1.03% by 2022) [17,18]. This trend of recovery indicates the strong resilience of the Japanese economy.

Furthermore, it also changed people’s behavior, such as following the advisory to stay at home, as confirmed by cell phone location data [19-21]. Such large-scale societal and behavioral changes warrant further investigation through various means to offer a chance to monitor and reflect the short- and long-term impacts of COVID-19 in the future.

### Literature Review

The disruption caused by pandemic-related restrictions may be seen as a failure to perform planned activities, but detecting such disruptions was challenging. For example, it is difficult to obtain behavioral data on trips that individuals could not take or events they could not attend owing to the restriction. Social media, which people use to share their activities, proved to be a great source of information in such cases. Twitter (currently X) is a widely used social media platform in many countries and has a sufficiently large population for social data analysis in health care contexts [22,23]. Japan has a particularly high population density of Twitter users, even when compared to the major countries that use Twitter, such as the United States. Furthermore, owing to language exclusivity, it is easier to filter comments related to Japanese society using Japanese keywords [24]. Twitter has also been frequently used to help summarize peoples’ responses about the pandemic and its measures, showing the challenges experienced throughout [25]. Prior studies in Korea and Japan used Twitter to determine public opinion, showing popular words during the pandemic [26]. Because people actively share their daily lives on Twitter, the site has the potential to be a data source for investigating the impact of restrictions on the public. Using Twitter as a resource, this study aims to explore and visualize plans disrupted in Japan due to COVID-19 pandemic measures.

There are many studies on COVID-19 that investigate social media platforms, such as Twitter. Chen et al [27] investigated the levels of anxiety during the COVID-19 pandemic. The adverse effects on the mental health of the public was also one of the impacts of the pandemic, as explained in the research by Li et al [28], who analyzed COVID-19–related tweets into different emotions and investigated the mental health aspects and how they recovered from the COVID-19 crisis. Lyu et al [29] investigated the topics and sentiments in public COVID-19 vaccine–related discussions, whereas Krittanawong et al [30] investigated misinformation dissemination related to COVID-19 on Twitter. Aside from studies focused on the pandemic itself, COVID-19 vaccines have also been highly researched topics on Twitter. Ansari and Khan assessed public responses through sentiment analysis of COVID-19 vaccines using Twitter, revealing an overall negative tone in the tweets [31]. Ferawati et al [32] explored how Twitter reported vaccine-related side effects by comparing the side effects of 2 types of messenger RNA vaccines developed by Pfizer and Moderna in Japan and Indonesia, respectively. Gao et al [33] examined COVID-19 concerns in each Japanese prefecture, and Uehara et al [34] investigated the attitudes toward vaccines or vaccination during the COVID-19 pandemic in different Japanese prefectures using search queries from Yahoo! JAPAN. Our study adopts a unique approach to examine how the COVID-19 pandemic has disrupted everyday activities. Our main focus is on understanding the direct impact of the pandemic on society through the observation of expressions, life disruptions, and plans.

For research on citizen feedback, Ishida et al [35] proposed a method that uses social media data. They implemented a multitask learning framework to estimate the associated viewpoints using bidirectional encoder representations from the transformer model. However, this method requires considerable effort to label the data. This study uses search queries and validates word co-occurrence to infer the themes of topics discussed during the COVID-19 pandemic in Japan, proposing an efficient and low-resource method for social media analysis.

### Objectives and Approach

We aimed to report on the impact of COVID-19 on Japanese society by analyzing public opinions extracted from social media data. Specifically, we focused on the popular term *Corona no-sei* (in Japanese コロナのせい, meaning “due to COVID-19,” “because of COVID-19,” or “considering COVID-19”), which clearly conveyed complaints or concerns about life event disruptions caused by the COVID-19 pandemic. Our study used 2 types of data: the daily COVID-19 case count and Japanese tweets containing the Japanese phrase *Corona no-sei* posted on Twitter between February 1, 2020, and November 30, 2021. We analyzed the trends in the number of tweets and COVID-19 cases to quantitatively explore their relationship and the words frequently used in the tweets to qualitatively explore social needs in the first 2 years of the COVID-19 pandemic.

In conclusion, we critically compared our findings with those identified in other similar studies to provide an alternative evidence base for the impact of COVID-19 on Japanese society.

## Methods

### COVID-19 Cases

To track the daily rise in COVID-19 cases, we gathered the number of new positive cases in Japan by manually downloading data from a dedicated COVID-19 site maintained by the NHK, Japan’s national broadcaster [36]. Our aim was to investigate the correlation between the number of positive cases and the volume of tweets. A total of 1,726,943 COVID-19–positive cases were recorded between February 1, 2020, and November 30, 2021.

### Tweets and Keywords Extraction

Another data set for this study includes 300,778 tweets containing the Japanese phrase *Corona no-sei* during the same period as the recorded COVID-19 cases (between February 1, 2020, and November 30, 2021). We chose this period because by the end of January 2020, the Japanese government had officially established the Japan Anti-Coronavirus National Task Force to actively address the pandemic. In addition, we aimed to include the maximum possible data until the initiation of this study in mid-November 2021. Furthermore, this period also included 3 emergency announcements by the Japanese government, making it a representative period for studying the impact of COVID-19 on Japanese society.

We counted the number of tweets per month and found that the maximum number of tweets was 517,688 in April 2020; the minimum number of tweets was 24,625 in November 2021; and the average number of tweets was 136,717.6. The *Corona no-sei* phrase is frequently used by the public in social media and everyday conversation to express the (often negative) feelings when Japanese individuals’ activities or life plans were interrupted by the COVID-19 outbreak. Although there are several expressions synonymous with *Corona no-sei* (eg, “because of the new coronavirus” and “because of COVID-19”), we chose *Corona no-sei* as a casual expression used by the public in social media and colloquial speech. The tweet data were provided by the NTT DATA Corporation, which has a real-time backup of Japanese firehose data from X Corporation (formerly known as Twitter). Data access was granted to a few collaborative research institutes, including the University of Tokyo, and one of the authors was granted permission to use the self-adaptive classification system to extract the data and keywords [37].

Although applying a clustering approach for topic modeling can be useful in grasping the topics discussed in the tweets, it does not apply to our context wherein we were targeting *COVID-19* as the main subject and aiming to identify the co-occurrence of events. Instead, we extracted co-occurrence nouns and verbs from the obtained *Corona no-sei* tweets by applying dependency analysis implemented in the system developed by Yoshinaga et al [37-39]. We used the base-phrase chunker to extract all tweets containing the *Corona no-sei* keyword (“keyword” is *bunsetsu* in Japanese). The built-in classifier then extracted the relevant verbs, nouns, and verb-noun-pairs for users based on the nonstack dependency parser, which achieved 99.01% accuracy in base-phrase chunking and 92.23% accuracy in dependency parsing [37]. Researchers who did not use the system and database maintained by the University of Tokyo could use the same tool published by the laboratory Pecco and DepP [37-40]. To avoid overinterpretation, we omitted tweets that described a disruption of plans but did not include COVID-19–related keywords.

### Analysis of the Keywords and its Correlation to the COVID-19 Pandemic Trends

The contexts following *Corona no-sei*, which indicate a high level of negative concern about COVID-19, frequently contain verbs in the negative form and nouns associated with them. By aggregating these nouns and verbs, we extracted information on the restrictions imposed and the events or plans canceled owing to the COVID-19 pandemic. This information enabled us to capture the potential social and psychological impact of disrupted life plans. Note that, by events or plans, we refer to the specific *type of occurrences* (eg, university entrance exam) rather than a certain event (eg, a pop singer’s concert in 2019). The frequency of nouns and verbs in tweets containing *Corona no-sei* was counted to identify the restrictions placed on people’s lives.

To investigate the correlation between tweet volumes and COVID-19 cases, we constructed transition diagrams for each. In addition, Pearson correlation coefficients were also calculated. Next, we examined the nouns and verbs co-occurring with *Corona no-sei* over the entire study period and specifically on the day with the highest tweet activity.

The cross-validation of the keywords and tweet contents was performed by randomly extracting 20 tweets from the top 5 verb and noun pairs and other keyword pairs that were deemed worthy of discussion by the researchers. The tweet contents were further annotated to ensure that they were aligned with the researchers’ interpretations of keywords. We then discussed the themes extracted by analyzing and cross-validating the themes and noteworthy keywords.

### Ethical Considerations

This study used publicly available data and did not handle identifiable private information, meaning that it was exempt from Institutional Review Board approval according to the Ethical Guidelines for Research of the Japanese National Government [41]. The NTT DATA Corporation obtained tweets according to Twitter terms of service and approved the use of the data for this study.

## Results

**Figure 1:**
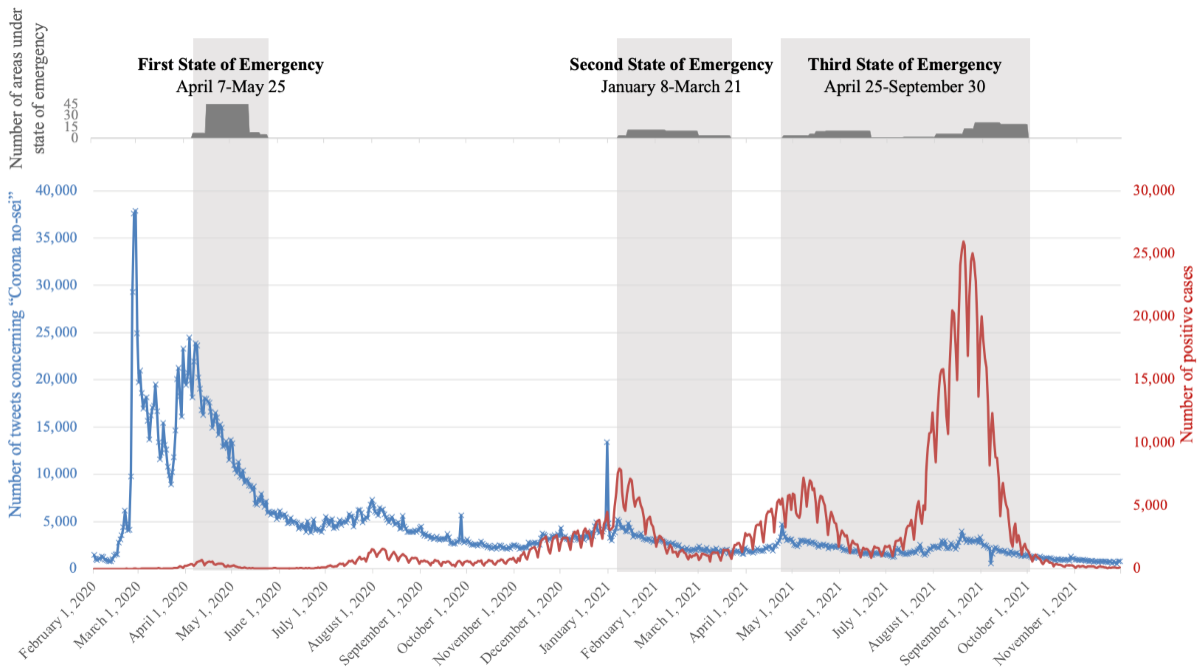
Trends in the number of *Corona no-sei* tweets and the number of patients with a COVID-19–positive result. The blue line indicates the number of *Corona no-sei* tweets, and the red line indicates the number of positive COVID-19 cases.

Figure 1 shows the time trend of *Corona no-sei* tweets (blue line) compared to the trend of positive cases (red line). There were 3 states of emergency announcements within our targeted period between February 1, 2020, and November 30, 2021, which are highlighted in gray in Figure 1. The number of areas under the state of emergency is indicated by the bar graph in the upper part of the figure because the target areas were changed during each state of emergency. The periods during which the states of emergency were imposed roughly corresponded to an increase in case numbers. Interestingly, the announcement of a state of emergency was highly effective in suppressing the number of cases. Regarding the spike caused by the Tokyo Olympics (which took place between July 23, 2021, and August 8, 2021), the case number quickly dropped to below 5000 per day within 3 months.

**Figure 2:**
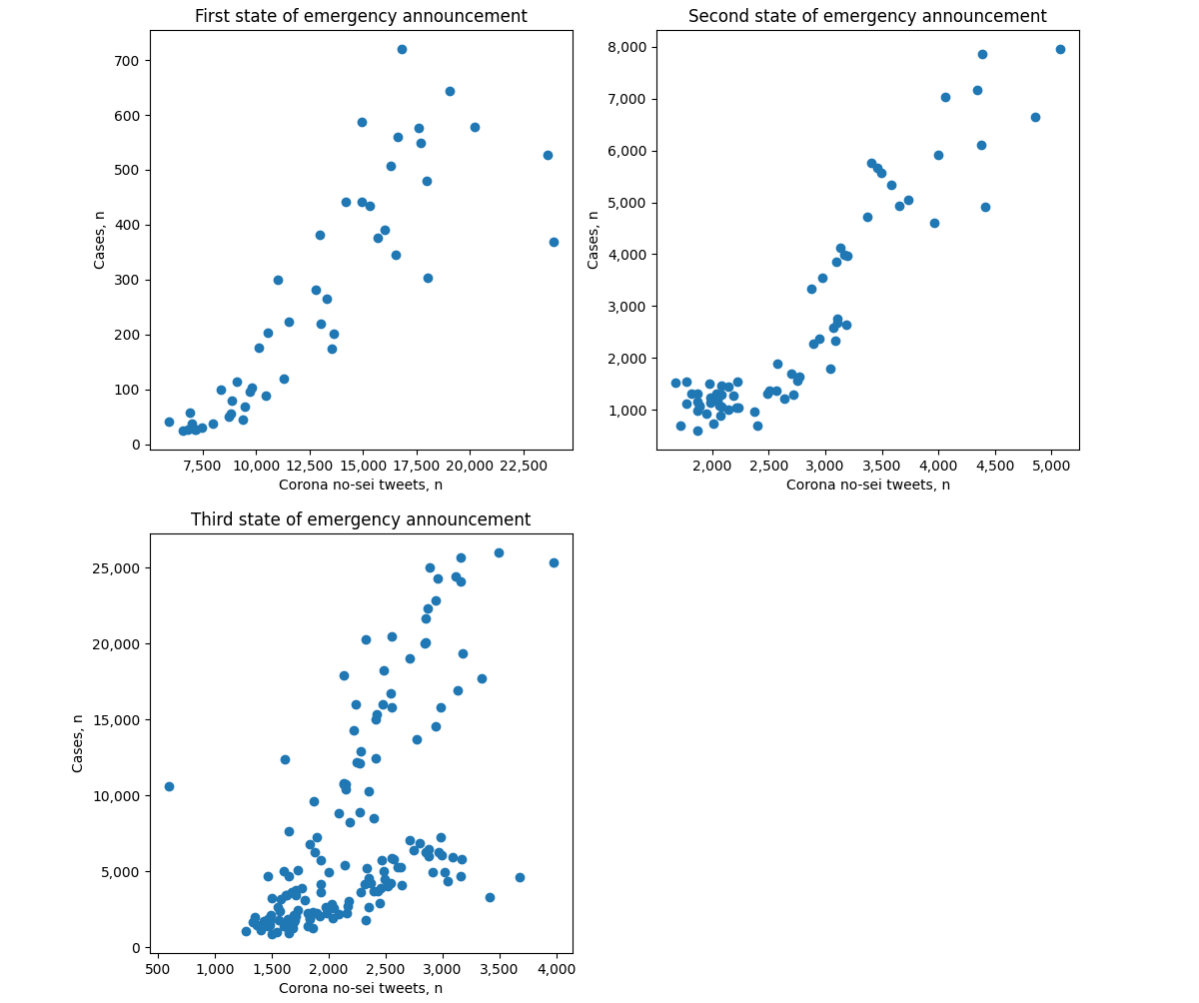
Scatter plot for COVID-19 case numbers and the number of *Corona no-sei* tweets for the first, second, and third state of emergency announcements.

As the blue line indicates, the *Corona no-sei* tweets peaked in March 2020, roughly before the first state of emergency was announced and reached the second highest number when the first state of emergency was imposed. After the first announcement of the state of emergency, the number of tweets using *Corona no-sei* showed a downward trend until the end of our data collection period. There were a few instances of small increases in *Corona no-sei* tweets before the second and third states of emergency announcements, but overall, the number of reported plan disruptions never reached the level observed before the first state of emergency announcement. The scatter plot for case numbers and the numbers of *Corona no-sei* tweets is shown in Figure 2, with Pearson correlation coefficients of 0.86, 0.93, and 0.61, respectively, for the first, second, and third states of emergency.

When compared with the number of the *Corona no-sei* tweets during the entire period, the correlation between COVID-19 daily cases and the *Corona no-sei* was not very evident. We were able to observe a slight increase of *Corona no-sei* tweets before the case number started rising, but the extent of increase in case numbers was disproportional to the extent of increase in *Corona no-sei* tweets. Even though the number of cases peaked in September 2021 during the third state of emergency, there was only a slight increase in *Corona no-sei* tweets compared to the high number of complaints at the very beginning of the COVID-19 pandemic. This indicates that Japanese residents might have adapted to the restrictions or disruptions caused by the COVID-19 pandemic lockdown.

We further investigated the nouns and verbs in the tweets that we sampled. Tables 1 and 2 show the number of tweets for the top 20 nouns and verbs tweeted on February 28, 2020, when the tweet number reached the highest level. Tables 3 and 4 show the top 20 words (nouns and verbs) that co-occurred with *Corona no-sei* tweets in descending order to highlight the most disrupted activities or plans during our data aggregation period. For nouns, here, *Corona* was excluded because it was a word included in the query and was clearly the most frequently detected. For nouns, the top 5 most frequently mentioned words were *work*, *abort*, *home*, *live*, and *friends* after excluding the words that indicate the grammatical tense. These keywords indicated that, over a longer period, Japanese individuals started developing concerns over their disrupted work and social life. For verbs, *go* was the most frequent, but in the actual tweets, it was sometimes used in the negative, and in the context, the verb was unlikely to be used in the affirmative, so the verb was likely used to indicate they *cannot go* even if it is in the affirmative in this paper (Textbox 1). Hence, the top 5 mean *go*, *can go* <negation>, *look*, *meet* <negation>, and *get out*. The results show that there are restrictions on the actions of going, seeing, and meeting as verbs. Compared with the single-day result on February 28, 2020, the concern about work appeared as the top 5 in Tables 1 and 2, suggesting that Japanese individuals placed clear emphasis on their work routines. In addition, the desire for live concerts increased over the long run, making *live concert* the fourth most frequently mentioned keyword in Table 1. Coincidentally, concerns related to friends and missing opportunities to meet up were also observed in both tables, showing the disruption of social relationships and recreational occasions. Both studies indicated that people regarded the COVID-19 pandemic as the main cause of their disrupted plans to hang out with friends or attend large public events. In addition to activities, the keyword finding also reflected the concern of resource shortage, such as toilet paper, masks, and even money, which were critical in supporting daily lives or normal health care practices.

Because the keywords indicated both long-term and short-term concerns, we cross-validated the tweet contents by selecting keyword pairs based on the top 5 keywords related to long-term concerns and those related to short-term anxiety on material shortage. A total of 160 tweets were randomly sampled based on the following keyword pairs (20 tweets each): ライブ＋行く／行けない (*live concert*+*go*/*go*<negation>); 家＋行く／行けない (home+go/go<negation>); 友達＋行く／行けない (*friends*+*go*/*go*<negation>); 中止＋行く／行けない (*cancel*+*go*/*go*<negation>); 友達＋会う／会える (friends+meet/can meet); 仕事＋行く／行けない (*work*+*go*/*go*<negation>); トイレットペーパー＋なくなる (toilet paper+vanish); and マスク＋なくなる (*mask*+*vanish*). One of the authors annotated the tweets according to the themes reflected by the keywords. Key findings are discussed in the following section.

**Table 1:** The number of tweets with co-occurring nouns on February 28, 2020.

Noun	Tweets, n (%)
graduation ceremony	2154 (14.55)
cancel	1813 (12.25)
school	1498 (10.12)
work	1210 (8.17)
event	775 (5.24)
live concert	694 (4.69)
toilet paper	667 (4.51)
part-time job	639 (4.32)
school holiday	636 (4.3)
friends	616 (4.16)
disney	605 (4.09)
postponement	562 (3.8)
home	553 (3.74)
mask	531 (3.59)
new corona	330 (2.23)
test	328 (2.22)
tissue	321 (2.17)
hoax	318 (2.15)
company	304 (2.05)
next month	250 (1.69)

**Table 2:** The number of tweets with co-occurring verbs on February 28, 2020.

Verb	Tweets, n (%)
disappear	3265 (20.15)
go	1835 (11.33)
rest	1169 (7.22)
can go <negation>	1044 (6.44)
end	862 (5.32)
go out	754 (4.65)
look	734 (4.53)
make effort	711 (4.39)
buy	602 (3.72)
cry	576 (3.56)
vanish	556 (3.43)
can meet <negation>	524 (3.23)
play	505 (3.12)
spring rest	501 (3.09)
dies	499 (3.08)
be told	491 (3.03)
come	441 (2.72)
think	436 (2.69)
return	378 (2.33)
crush	319 (1.97)

**Table 3:** Noun words co-occurring with *Corona no-sei* in descending order.

Order	Noun
1	work
2	cancel
3	home
4	live concert
5	friends
6	event
7	postponement
8	stress
9	school
10	part-time job
11	company
12	new corona
13	mask
14	graduation ceremony
15	hospital
16	one person
17	opportunity
18	university
19	family
20	money

**Table 4:** Verb words co-occurring with *Corona no-sei* in descending order.

Order	Verb
1	go
2	can go <negation>
3	look
4	meet <negation>
5	get out
6	lose
7	make effort
8	increase
9	buy
10	lose
11	end
12	come
13	think
14	meet
15	rest
16	can go
17	decrease
18	be told
19	meet
20	play

**Examples of tweets posted on Twitter (Japanese tweets were translated into English).**

**Verb and example**

- Go: “Due to COVID-19, the day I've been looking forward to going out with the guy I love has been postponed... I can't help it now and I'll accept it, but I was looking forward to it.”
- Meet: “It doesn't feel like April at all due to COVID-19, but I can't wait for it to end so that we can all meet, eat, and shop together comfortably. Six years already... I want to quit my job lol.”

## Discussion

### Principal Findings

Our findings revealed that the COVID-19 pandemic significantly disrupted daily routines in Japan, particularly in terms of work, education, social activities, and material shortages (with regard to the temporary spike of anxiety). The findings from our study correspond with numerous studies conducted in diverse countries, highlighting the extensive impact of the COVID-19 pandemic on social life, economy, public mental health, and education [5]. In this section, we discuss key findings across a temporal spectrum, focusing on 4 crucial aspects: disruption of work routines, public anxiety stemming from perceived resource shortages, concerns regarding social relationships, and interference with the curriculum.

### Top Concerns

The impact of the COVID-19 pandemic on the labor market in Japan is unequivocal, mirroring the challenges faced by numerous countries. The pandemic necessitated a shift in work dynamics with the unintended pilot of remote collaboration. Notably, certain categories of Japanese workers, contingent on their employment contracts, exhibited heightened susceptibility to these alterations in work patterns. In our findings, the keyword *work* demonstrated associations with *part-time*, *abort*, and *money*, indicating that individuals expressing concerns about their work conditions may grapple with job uncertainty, stemming either from the part-time nature of their employment or an abrupt reduction in income. This discovery aligns seamlessly with prior research examining the repercussions of the COVID-19 pandemic on Japan’s labor sector. As described by Kikuchi et al [42] in their study, individuals in contingent employment, along with women and those with lower income, were notably susceptible. The shift toward teleworking and the accompanying uncertainty about long-term income during the COVID-19 pandemic had a disproportionately adverse impact on these specific demographic groups [42]. Fukai et al [43] endorsed these findings through extensive government statistical analysis. According to their research, Japanese individuals employed part-time in service industries or compelled to take leave or face job loss following the declaration of a state of emergency were identified as particularly vulnerable groups significantly affected by the COVID-19 pandemic [43]. Although the use of part-time or contingent workers has traditionally been a standard practice for Japanese companies seeking to optimize budget and resource allocation, the advent of the COVID-19 pandemic has pushed issues related to *work* to the forefront of public concern. Researchers caution that this could potentially exacerbate inequality for susceptible individuals unless actively addressed by government support [44].

In summary, our findings provide substantial evidence for concerns among Japanese internet users regarding job disruption, employment disparities, and inadequate financial resilience. Failing to address these issues during multiple states of emergency, the Japanese government risks compromising the equality within Japan’s labor market significantly. Interestingly, a study conducted by Chen et al [27], who sampled 6535 Reddit posts, identified strikingly identical subjects that propelled nationwide anxiety in the United States. Notably, concerns about career, finance, and the future were prevalent. However, our research suggests that health and death concerns were not as prominent in Japan, as observed in the study by Chen et al [27]. We hypothesized that the emphasis on collectivism and harmony in Japanese society could shape individuals’ concerns during crises (particularly in the case of a national crisis). For example, apprehensions about not being perceived as “useful” or causing “inconvenience” to others, possibly even relying on government subsidies, were more pronounced than concerns related to health and mortality.

### Sudden and Perhaps Excessive Anxiety About Material Shortage

The scarcity of certain items, including toilet paper, masks, and tissues, as outlined in Table 1, emerged as a significant issue in Japan. Our findings closely parallel earlier Twitter studies investigating hoarding behaviors, particularly concerning toilet paper [45]. Although initially observed in the United States, panic buying for household goods rapidly became a global phenomenon. Notably, toilet paper has emerged as a frequently hoarded item, often signaling a surge in demand during natural disasters [46,47]. Although the act of stockpiling toilet paper may seem irrational and has been widely ridiculed on social media, the adverse effects of bulk purchasing have not been as severe. Social scientists may view this behavior as a coping mechanism during a natural disaster [48]. Contrary to the commonly perceived overhoarding of toilet paper, the mask shortage was deemed a more severe public health crisis and a direct threat to well-being. A 2020 agent-based simulation conducted by Tatapudi et al [48] illustrated that universal mask use could potentially reduce infections by 20% [49]. At the time of the study, the total number of people infected by COVID-19 was 541 million, indicating that implementing universal mask use could potentially spare 108 million cases. Numerous studies have indicated a negative correlation between mask use and the COVID-19 infection rate [44,50].

However, the situation in Japan presents a slightly different scenario. The Japanese government faced criticism for a perceived slow response to the awareness of mask shortages, as the pandemic was considered relatively “under control” in its early stages. As the mask crisis unfolded, many Japanese citizens became concerned about their reliance on masks manufactured abroad, prompting the government to take actions to boost domestic mask production. Unfortunately, heightened anxiety also led to the “Abenomasks” incident, wherein the government faced backlash for stockpiling over 82 million unused masks [51]. A crucial lesson learned from this incident is that although social media serves as a critical channel for the dissemination of news and raising public awareness, the emotional contagion and overpromotion of a particular disaster can backfire, impeding the rational coping mechanisms of citizens and the decision-making processes of the government. Our findings, along with those of numerous other studies, indicate that further efforts are needed to develop effective protocols for addressing the widely contagious anxiety stemming from the dissemination of information about natural disasters on social media.

### Concerns About Social Relationships

Keywords pertaining to relationships, social life, and collective events were prevalent in our analysis. For instance, the top 20 frequently occurring nouns associated with *Corona no-sei* included friends, family, live, events, and one person. The most frequent verbs in the context of *Corona no-sei* were *go*, *can go* <negation>, *meet* <negation>, *buy*, *meet*, *can go*, and *play*. The example in Table 4 illustrates how Japanese individuals linked *go* and *meet* to their social events. While it may appear that many tweets express concerns about social relationships, these keywords actually reflect people venting their frustration about being unable to meet and engage in activities together, rather than indicating an actual loss of relationships. Interestingly, a study by Goodwin and Takahashi [52] also yielded similar findings. Most Japanese respondents in their survey gauging perceptions of relationship quality during the COVID-19 pandemic indicated that there were no discernible changes in their perceived relationship quality. Only a few reported that their trust and relationship with communities had declined compared to the prepandemic era [52]. There was also a report indicating that students, due to reduced communication with friends, face an increased risk of mental health problems [53].

These findings suggest that events, such as the COVID-19 pandemic, may lead individuals to experience heightened anxiety and stress. While this emotional response could temporarily disrupt their social activities and coping mechanisms against trauma, it may not have a lasting impact on their perceived relationship quality. In fact, the example tweets we analyzed illustrated how individuals, despite feeling frustrated, expressed eagerness to resume their social activities after the pandemic. Hence, we argue that concerns about relationship disruption may be transient and serve as a positive signal prompting individuals in Japan to actively nurture their relationships. As suggested by the study conducted by Goodwin and Takahashi [52], dedicating additional time to communication, particularly in the context of romantic relationships, could further enhance the quality of these connections [42].

### Concern for Education Discontinuation

The peak volume of tweets was recorded on February 28, 2020, coinciding with the government’s announcement of the simultaneous closure of all elementary, junior high, and senior high schools in Japan. In fact, in the most frequent nouns and verbs shown in Tables 1 and 2, the top words related to the simultaneous closure of schools were *graduation ceremony*, *cancel*, *lose*, *rest*, and *go* <negation>, all of which reflected Japanese citizens’ concerns about the discontinuation of education, the cancelation of the graduation ceremony, and missing school classes. It is essential to note that in Japan, the graduation ceremony typically takes place in March and the new school and work year commences in April. Despite the Japanese government’s earnest efforts to mitigate the spread of COVID-19, as scrutinized by scientists, the decision to close schools in Japan did not yield a substantial impact on preventing the spread of COVID-19. Instead, it deprived children of valuable learning and developmental opportunities [54]. Moreover, with the closure of schools, there was a surge in the demand for digital education or internet-based learning platforms. However, many schools and student households were ill-equipped to handle this impromptu shift to an internet-based education system. As discussed in detail by Iwabuchi et al [55], the unequal distribution of resources among schools in Japan further intensified the digital learning disparities brought about by the COVID-19 pandemic–induced school closures. The more well-funded private and prefecture-sponsored schools had often already implemented or could quickly set up the necessary e-learning system to cope with the lack of face-to-face lecturing. However, most public schools were forced to send learning materials to students by mail, risking a huge learning gap between students in private and public schools. The long-term impact on students’ physical and mental development remains uncertain, given that most schools were able to resume normalcy after the lifting of the state of emergency. A study conducted by Nishimura et al [56] on medical students clearly indicated a deterioration in subjective mental well-being.

Concerns were also observed regarding web-based alternatives, with growing apprehensions that they fail to adequately substitute the essential in-person learning and hands-on field practice integral to medical education. The diverse concerns reflected in education-related keywords in Table 1 suggest that many Japanese individuals transitioned their focus from one-time events, such as *graduation ceremony* and *school holiday*, to longer-term mental and societal impacts, such as *opportunity*, *stress*, and *university*. This shift implies that the long-term effects would take time to manifest compared to short-term disruptions of specific incidents, such as a graduation ceremony. Further studies are crucial to monitor and unveil a complete picture of this disruption.

### Long- and Short-Term Concerns and the Impact on the Society

Following the World Health Organization’s official declaration that COVID-19 was no longer considered a global health emergency on May 4, 2023, individuals who survived now faced a familiar daily life with some changes that were difficult to imagine in the prepandemic era. However, there is still an impact on society that can be challenging to trace and monitor. The economic repercussion, such as inflation and tumbling currency values in Japan, are gradually occurring. Schoolchildren who have lost education for almost 1 year are bracing for their future growth. An increasing number of companies are eager to get talent to opt in for remote working styles to attract employees who were reluctant to return to city offices. Individuals are probably no longer worried about toilet paper but will gradually sense the subtle shifting of their workstyles, social styles, and even learning styles. However, due to the limitations of our data, we were not able to speculate about the postpandemic future. Our discussion offers possible clues to further trace the causes of societal changes. The profound effects of the COVID-19 pandemic on society and public health require further investigation and monitoring.

### Limitations and Future Work

It should be noted that our study had some limitations in extracting data from social platforms such as Twitter. One limitation is the lack of geolocation metadata. Although we capitalized on the language exclusivity of Japanese tweets and the domestic majority to extract representative samples of Japanese citizens, it is important to note that there may be some minor contributions from Japanese speakers residing outside Japan. This limitation should be considered when interpreting the findings of this study. Another limitation arises from the bias present on Twitter, as its use is lower among older adults compared to the younger population. To mitigate this bias, stratified analysis is necessary to account for the effects of age. However, the current system lacks age data. Consequently, the results should be interpreted with the awareness that the perspectives of the older adults are underrepresented.

Because the purpose of this study was to derive an interruption schedule, we specifically targeted verbs and nouns to better represent social connections (families and friends), locations, events, subjects, and actions, rather than using adjectives or phrases that might focus on emotional descriptions or concrete situations. This approach limited our options for sentiment-related analysis methods or topic modeling, which could reveal emotional reactions instead of generic events and the involvement of close connections. Although people’s sentiments were deemed beyond the scope of this study, in future studies, we would like to analyze how people’s sentiments have changed through sentiment analysis [57]. With the introduction of transformer-based large language models, such as bidirectional encoder representations from transformers and text-to-text transfer transformers, more contextual and in-depth understanding and analysis might be made available for researchers in social media data. This should be considered in future studies.

We also did not address concerns regarding the safety of cybersecurity during the work-from-home period during the pandemic. We noticed that in the United States, data breaches and the security of the work environment were one of the top concerns [58]; however, based on our current results, there was no direct implication on this aspect in Japan during the COVID-19 pandemic. This will be considered in our future work.

### Conclusions

Overall, by adding the analysis on *Corona no-sei* to the conventional symptom-based monitoring, we were able to identify the underlying concerns at the peak of the disruption and across the whole-time span of the 3 announcements of state of emergency. Our findings and a comparison of the tweets against COVID-19 case numbers yielded rich insights into people’s short- and long-term concerns and potential aspects of societal impact caused by the announcements of the state of emergency. Although more studies from different fields would help to reveal the whole landscape of social and psychological impact caused by COVID-19, we believed that the keywords reflected in *Corona no-sei* tweets provided more nuanced descriptions of real-life problems Japanese individuals faced during the COVID-19 pandemic and revealed the development of different concerns in response to the change of policies.

Timely communication of analysis results is crucial, especially when dealing with issues of significant social impact, such as a global pandemic. A delay in delivering results can hinder decision-making processes and require substantial resources to recover from the initial losses caused by poor decisions. For policy makers, especially the Japanese government, this study reflects the opinions of citizens and should be considered when reviewing the effectiveness and suitability of a policy as well as assessing further measures to support those impacted during the pandemic.
