# Correction: Exploring the Impact of the COVID-19 Pandemic on Twitter in Japan: Qualitative Analysis of Disrupted Plans and Consequences

**DOI:** 10.2196/67434

**Published:** 2024-10-29

**Authors:** Masaru Kamba, Wan Jou She, Kiki Ferawati, Shoko Wakamiya, Eiji Aramaki

**Affiliations:** 1 Division of Information Science Graduate School of Science and Technology Nara Institute of Science and Technology Ikoma Japan; 2 Institute of Industrial Science The University of Tokyo Tokyo Japan

In “Exploring the Impact of the COVID-19 Pandemic on Twitter in Japan: Qualitative Analysis of Disrupted Plans and Consequences” (JMIR Infodemiology 2024;4:e49699) the authors made one addition.

The fourth author was listed as follows:

Shoko Wakamiya^1^, PhD.

1. Division of Information Science, Graduate School of Science and Technology, Nara Institute of Science and Technology, Ikoma, Japan

This has been changed by adding the second affiliation as follows:

Shoko Wakamiya^1, 2^, PhD.

1. Division of Information Science, Graduate School of Science and Technology, Nara Institute of Science and Technology, Ikoma, Japan

2. Institute of Industrial Science, The University of Tokyo, Tokyo, Japan

The correction will appear in the online version of the paper on the JMIR Publications website on October 29, 2024, together with the publication of this correction notice. Because this was made after submission to PubMed, PubMed Central, and other full-text repositories, the corrected article has also been resubmitted to those repositories.

